# Improving lymphopoiesis in aged bone marrow

**DOI:** 10.1097/MOH.0000000000000923

**Published:** 2026-03-18

**Authors:** Anna Konturek-Ciesla, David Bryder

**Affiliations:** a Department of Biosystems Science and Engineering, ETH Zurich, Basel, Switzerland; b Division of Molecular Hematology, Lund Stem Cell Center, Lund University, Sweden

**Keywords:** hematopoietic stem and progenitor cells, immune aging, lymphopoiesis, regeneration

## Abstract

**Purpose of review:**

Aging is associated with impaired B lymphopoiesis and T lymphopoiesis, contributing to immunosenescence and poor immune recovery. Although this decline can be attributed to intrinsic hematopoietic stem cell aging, growing evidence indicates that lymphoid failure reflects constraints operating across multiple levels of the hematopoietic system. This review frames age-associated lymphopoiesis decline as a systems-level problem and outlines conceptual avenues for therapeutic intervention.

**Recent findings:**

Age-associated lymphoid failure is increasingly attributed to inflammatory suppression, dominance of dysfunctional stem and progenitor states, and compromised extramedullary support. These insights provide a framework for interventions that restore immune competence by rebalancing hematopoiesis or selectively replacing compromised stem cell function.

**Summary:**

Age-associated lymphoid decline arises from coordinated constraints across the bone marrow niche, stem and progenitor composition, and extramedullary lymphoid support, rather than intrinsic stem cell exhaustion alone. Targeting these bottlenecks in a context-dependent manner offers multiple routes to improve lymphopoiesis and restore immune competence in aging.

## INTRODUCTION

Aging is associated with a progressive decline in B lymphopoiesis and T lymphopoiesis, contributing to immunosenescence, impaired vaccine responses, and increased susceptibility to infection and malignancy [1]. Early conceptual models attributed this primarily to intrinsic aging of hematopoietic stem cells (HSCs), based largely on transplantation studies showing reduced lymphoid output and an apparent shift toward myeloid differentiation [2]. This view gave rise to the prevailing idea that aged HSCs become intrinsically ‘myeloid-biased’ [3].

However, lymphoid output depends on multiple sequential bottlenecks rather than a single lineage decision. In the bone marrow, hematopoietic stem and progenitor cells (HSPCs) must generate lymphoid-primed intermediates to sustain B-cell production. In parallel, T-cell development requires effective generation of thymus-seeding progenitors and adequate extramedullary support. Recent studies support a model in which age-associated hematopoietic skewing reflects changes in HSPC composition, marked by loss of short-term, lymphoid-contributing HSCs, early lymphoid-primed progenitors [4,5], and thymic early T-cell progenitors [6]. Notably, platelet-biased HSCs increase with age and may further dilute effective lymphoid output [7], reinforcing the appearance of lineage skewing without requiring irreversible loss of lymphoid competence at the HSC level. Consistent with this view, native lineage tracing and multimodal profiling studies place the principal lymphoid bottleneck at early differentiation steps downstream of HSCs, where lymphoid-primed progenitors are disproportionately depleted with age [5,8]. 

**Box 1:**
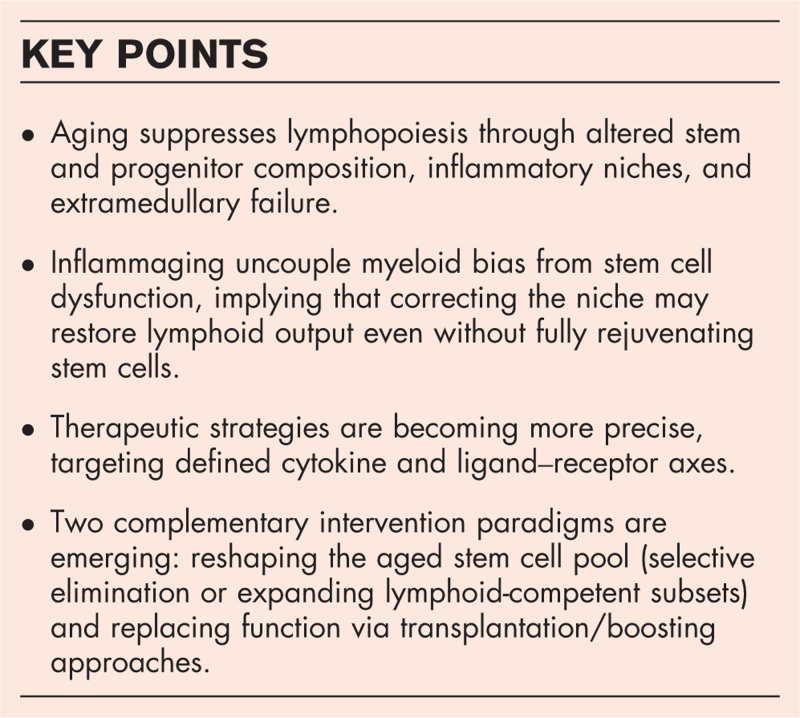
no caption available

**FIGURE 1:**
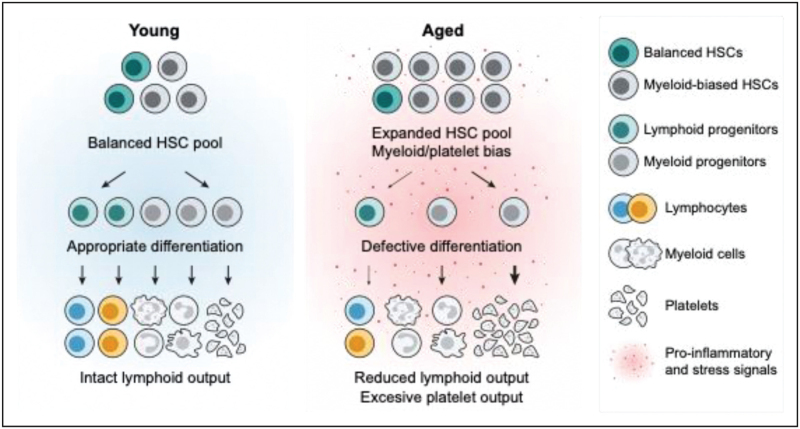
Age-associated constraints on lymphoid output across the hematopoietic hierarchy. In young bone marrow (left), a balanced hematopoietic stem cell (HSC) pool supports efficient generation of appropriately specified progenitors and robust lymphoid output. With aging (right), expansion of dysfunctional HSC states, including myeloid-biased and platelet-biased and senescent populations, alters early differentiation dynamics. These changes, together with inflammatory niche stress, impair progenitor cell differentiation, leading to reduced lymphoid output and a relative expansion of myeloid lineages.

Together, these findings help to reframe age-associated lymphopoiesis failure as arising from distributed constraints across HSC composition, early progenitor differentiation, and extramedullary lymphoid support, rather than from an exclusive intrinsic fate switch in aged HSCs (Fig. 1).

## INFLAMMAGING AND NICHE SIGNALING AS SUPPRESSORS OF LYMPHOID OUTPUT

Chronic, low-grade inflammation is recognized as a dominant constraint on lymphoid output during aging [9] (Fig. 1). One mechanistic link between inflammaging and HSC dysfunction involves immune-complex sensing through Fc receptor pathways. BCL11A, classically associated with lymphoid development, has emerged as a context-dependent regulator of inflammatory tone in hematopoiesis [10]. In HSCs, BCL11A represses FCER1G expression, restraining inflammatory signaling and preserving quiescence and self-renewal. In contrast, BCL11A activity in myeloid cells promotes IL-1β production, indirectly impairing HSC function through niche-derived inflammatory cues. This IL-1β–FCER1G axis provides a conceptual framework linking immune-complex signaling to cytokine-driven suppression of lymphoid competence.

Inflammatory niches also shape lineage output through compartment-specific activation of NF-κB signaling. Experimental dissection of NF-κB activity demonstrates that inflammatory signaling within the bone marrow milieu is sufficient to impose myeloid priming and suppress lymphoid output from otherwise normal HSPCs [11]. By contrast, HSC-intrinsic NF-κB activation is associated primarily with deeper quiescence and impaired regenerative capacity rather than overt lineage skewing. These findings directly uncouple myeloid bias from intrinsic HSC dysfunction and position the inflammatory niche as a dominant determinant of lineage output.

Importantly, inflammatory signaling need not act exclusively at the level of HSC fate (Fig. 1). Mathematical modeling and single-cell analyses indicate that chronic NF-κB activity reshapes hematopoiesis by simultaneously reducing lymphoid branching and expanding myeloid-primed progenitor populations downstream of HSCs [12]. This combination is sufficient to reproduce aging-like output patterns, underscoring how progenitor population dynamics can impose lymphoid failure independent of HSCs.

Beyond cytokine signaling, aging alters specialized niche components that normally enforce HSC restraint. The megakaryocyte-vascular niche has emerged as a key inflammation-sensitive regulator of lymphoid output. Age-associated remodeling of megakaryocytes is accompanied by reduced production of platelet factor 4 (PF4/CXCL4), a niche-derived chemokine that promotes HSC quiescence and functional integrity [13]. Loss of PF4 phenocopies premature hematopoietic aging, whereas restoring PF4 in aged mice improves HSC function and rebalances lineage output, including enhanced lymphoid potential. The conserved responsiveness of human CD34^+^ HSCs to PF4 underscores the translational relevance of this axis.

Systemic inflammatory mediators further reinforce niche dysfunction. Thrombospondin-1 (THBS1), elevated in aged plasma, induces a conserved HSC inflammaging transcriptional program through CD36-dependent signaling [14]. Genetic deletion of THBS1 preserves HSC function, limits myeloid skewing, and improves hematopoietic health span, linking systemic inflammatory cues to local niche remodeling and lymphoid suppression.

Inflammation also stabilizes maladaptive HSC states through altered adhesion and trafficking cues. Inflammatory stimuli induce SELP (P-selectin) expression in HSCs, and sustained SELP overexpression amplifies inflammatory signaling via IFNγ-dependent PI3K–AKT–mTOR pathways [15]. SELP^HI^ HSCs accumulate with age and represent a low-fitness, myeloid-biased and platelet-biased subset whose transcriptional identity is strongly shaped by the niche [16]. Engagement of SELP by its ligand PSGL-1 can dampen inflammatory signaling and partially restore youthful transcriptional programs, whereas reduced PSGL-1 availability in the aged niche may lock HSCs into an inflammaging state.

Finally, age-associated inflammatory remodeling perturbs Notch signaling, a pathway essential for early lymphoid specification. Reduced expression of the Notch ligand Jagged2 in the bone marrow, together with a shift from trans-activation to cis-inhibition in aged HSCs, promotes myeloid bias and diminished regenerative capacity [17]. In parallel, impaired Notch signaling in bone marrow and thymic microenvironments limits the generation and thymic seeding of early lymphoid progenitors, contributing to the early decline in T-cell output [6].

At the same time, accurate interpretation of inflammatory states is essential for translating these insights into actionable strategies. Transcriptional signatures commonly associated with inflammation, including immediate early response gene programs, can be rapidly induced by ex-vivo handling in both young and aged HSCs and therefore do not necessarily reflect bona fide age-dependent niche inflammation [18^▪^]. Accordingly, rigorous control of tissue handling, sampling context, and comparative baselines is critical, particularly in human studies where material is limited, and processing-induced stress is difficult to avoid.

**FIGURE 2:**
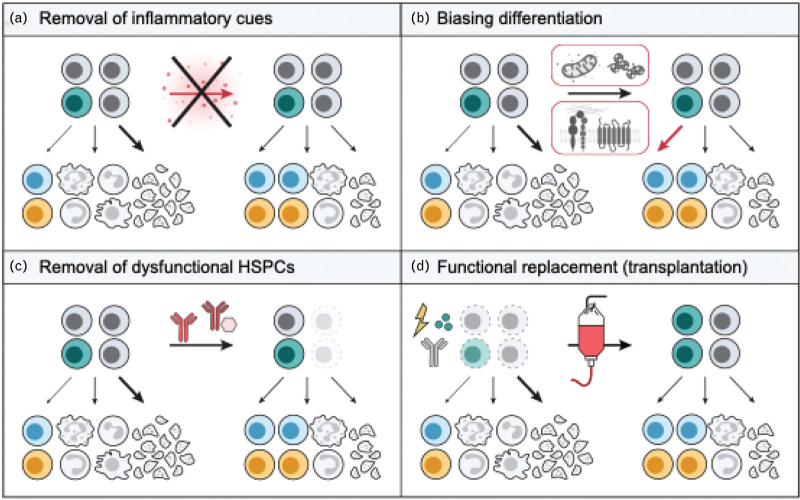
Therapeutic strategies to restore lymphoid output in aged hematopoiesis. Emerging interventions aimed at improving lymphopoiesis in aged bone marrow. (a) Targeting niche-derived inflammatory and stress signals alleviates extrinsic suppression of lymphoid differentiation. (b) Modulation of mitochondrial activity, chromatin states, cell adhesion, and mechanical sensing promotes lymphoid-primed differentiation at the stem/progenitor level, enhancing lymphoid cell generation despite persistent aging features. (c) Targeted depletion of senescent or lineage-biased hematopoietic stem and progenitor cell (HSPC) subsets reshapes the hematopoietic stem cell (HSC) pool and rebalances downstream lineage output. (d) Nongenotoxic conditioning followed by transplantation or boosting with lymphoid-competent HSCs restores immune cell production when endogenous repair mechanisms are insufficient. Together, these approaches illustrate a shift from global rejuvenation toward context-aware strategies that selectively relieve dominant barriers to lymphopoiesis.

Collectively, these findings converge on a model in which inflammaging suppresses lymphopoiesis through coordinated effects on cytokine signaling, immune-complex sensing, progenitor population dynamics, niche-derived restraint signals, adhesion-mediated feedback, and developmental signaling pathways. Importantly, many of these constraints appear at least partially reversible (Fig. 2), positioning the aged inflammatory niche as a central and potentially tractable therapeutic target for restoring lymphoid output.

## HEMATOPOIETIC STEM CELL HETEROGENEITY: IDENTIFYING AND MANAGING ‘BAD ACTORS’

Age-associated hematopoietic dysfunction is increasingly understood as a problem of composition rather than uniform decline (Fig. 1). Rather than affecting all HSPCs equally, aging is accompanied by the disproportionate expansion of discrete HSPC states with reduced lymphoid competence and impaired regenerative capacity [19]. This shift in perspective has moved the field away from attempts at system-wide rejuvenation and toward strategies that selectively limit, counterbalance, or remove dominant dysfunctional contributors to aged hematopoiesis.

This principle is well illustrated by studies targeting CD150^HI^ HSCs, a subset enriched for myeloid-biased and platelet-biased differentiation and poor lymphoid output in aged mice [19,20]. Restricting the contribution of this compartment alleviates aging-associated phenotypes in transplantation settings [21^▪^], and antibody-mediated depletion of CD150^HI^ HSCs reshapes the aged HSC pool *in situ*, reducing myeloid skewing and improving lymphoid output [22]. Related antibody-based approaches targeting myeloid-biased HSC states reinforce the concept that selective removal of disproportionately maladaptive subsets can rebalance hematopoietic output and improve immune function in aged hosts [23^▪▪^] (Fig. 2).

Human studies provide complementary support for this framework. Single-cell analyses of aged human bone marrow have identified a senescent HSPC population marked by telomere damage, activation of a TP53-CDKN1A (p53-p21) axis, and cell-cycle arrest [24]. Importantly, this senescent program appears largely intrinsic and precedes overt inflammatory remodeling, which emerges primarily in downstream progenitors. These observations suggest that age-associated dysfunction can originate within specific HSPC compartments and raise the possibility that selectively eliminating TP53-CDKN1A-positive states may represent a tractable form of senolytic intervention (Fig. 2), rather than necessitating indiscriminate targeting of the entire HSC pool.

At the same time, not all aged-associated states represent fixed or pathological endpoints. The identification of Clusterin (CLU) as a marker of aged-like HSCs highlights the need to distinguish stable maladaptive states from reversible stress-associated programs [7]. Although CLU^HI^ HSCs expand with age and exhibit reduced lymphoid output and myeloid/platelet bias [7,25], transplantation studies demonstrate substantial plasticity, with interconversion between CLU^LO^ and CLU^HI^ states. This plasticity implies that the durability of benefits achieved through selective elimination (Fig. 2) will depend on the surrounding niche context and on feedback mechanisms that govern re-emergence of dysfunctional states.

In parallel to depletion strategies, recent work has emphasized the therapeutic potential of stabilizing or amplifying lymphoid-competent subsets that persist with age (Fig. 2). HSCs with low KIT expression (KIT^LO^) display lymphoid-primed chromatin landscapes and superior B-cell and T-cell output in both mouse and human systems [26]. Although aging reduces the frequency of KIT^LO^ HSCs, remaining cells retain lymphoid competence, and enforced expression of the lymphoid regulator ZBTB1 can partially restore thymic reconstitution even in aged or lineage-biased contexts. Consistent with this, KITL expression by bone marrow mesenchymal stromal cells correlates with HSC activation state and lymphoid competence in middle-aged mice [27].

Finally, caution is warranted in assuming that all age-associated lineage biases are uniformly deleterious. Niche-derived signals such as SEMA4A actively preserve myeloid-biased HSC identity and functional resilience under inflammatory stress [28], indicating that certain biased states may confer adaptive advantages in aged or inflamed environments. More broadly, the timing and extent of immune aging vary substantially among individuals, reflecting heterogeneous aging trajectories of distinct HSC subsets [29]. From a therapeutic perspective, this argues against a requirement for full restoration of a youthful HSC state. Instead, selectively reinstating functions most directly linked to immune competence (Fig. 2), particularly effective lymphoid output, may yield substantial benefit even if other age-associated features persist.

## DIRECTLY BIASING AGED HEMATOPOIESIS TOWARD LYMPHOPOIESIS

Beyond identifying and managing dysfunctional HSC states, an emerging class of interventions aims to actively bias hematopoietic output toward lymphopoiesis or to restore function when endogenous repair is insufficient (Fig. 2). These strategies operate at complementary levels by tuning intrinsic metabolic and chromatin states that govern lineage competence and by modulating biophysical cues.

A central theme is the role of metabolic checkpoints in regulating HSC activation, exhaustion, and lineage output. The NAD+-consuming ectoenzyme CD38 becomes aberrantly upregulated in aged HSCs, promoting mitochondrial dysfunction, dysregulated calcium signaling, and reduced regenerative capacity [30]. Genetic or pharmacologic inhibition of CD38 preserves NAD+ availability, improves mitochondrial fitness, and restores more balanced lineage output in aged mice, including enhanced lymphoid contribution. Consistent with this principle, boosting mitochondrial quality through the Urolithin A similarly improves lymphoid compartments and strengthens antiviral immune responses in aged animals [31].

Metabolic dysregulation in aged HSCs also extends to lysosomal function. Aging is associated with hyperacidic lysosomes that impair cellular metabolism and reinforce inflammatory signaling [32^▪^]. Pharmacologic normalization of lysosomal activity partially restores youthful transcriptional states, dampens pro-inflammatory programs, and improves HSC function, including enhanced lymphoid potential.

Complementing metabolic control, chromatin architecture has emerged as a tunable regulator of lymphoid versus myeloid output. The linker histone H1.0 functions as a chromatin fate dial in HSPCs, with higher H1.0 levels restricting accessibility at myeloid-associated loci while preserving lymphoid programs [33]. Antagonism of H1.0 by HMGN1 shifts differentiation toward myelopoiesis, whereas maintaining H1.0 abundance promotes lymphoid differentiation without compromising HSC function. Importantly, inflammatory cues reduce H1.0 levels through protease-dependent mechanisms, suggesting that pharmacologic stabilization of H1.0, for example, via clinically tractable aspartyl protease inhibitors, could counteract inflammation-driven lymphoid suppression.

Beyond metabolic and chromatin control, biophysical cues represent an additional regulatory layer shaping age-associated HSC dysfunction. The mechanosensitive ion channel PIEZO1 promotes HSC proliferation and myeloid bias in response to shear stress and is upregulated in aged HSCs [34]. While PIEZO1 inhibition limits inflammation-associated aging phenotypes, it also compromises immune responses during acute infection, underscoring the trade-offs inherent in manipulating stress-adaptive pathways. In parallel, aged HSCs exhibit increased nuclear envelope tension, leading to activation of RHOA signaling [35^▪^]. Inhibition of RHOA restores youthful nuclear architecture, suppresses inflammatory transcriptional programs, and improves lineage-balanced output.

Collectively, these studies demonstrate that lymphoid decline with age is not a passive consequence of HSC attrition but reflects actively maintained states that can be modulated through metabolic, chromatin, and mechanotransductive pathways. Importantly, interventions at these levels bias hematopoiesis toward lymphopoiesis without requiring complete HSC replacement (Fig. 2), providing a mechanistically grounded framework for restoring immune competence while preserving essential stress-adaptive functions.

## NONGENOTOXIC REPLACEMENT FOR SYSTEM-LEVEL REJUVENATION

When intrinsic HSC repair or niche-directed modulation proves insufficient to restore lymphoid output, replacement strategies provide an alternative, system-level route to functional recovery. Antibody-based, nongenotoxic conditioning regimens that selectively deplete endogenous HSCs enable engraftment of transplanted HSCs without the toxicity associated with irradiation or chemotherapy [36^▪^] (Fig. 2). In aged recipients, transplantation of young HSCs following such conditioning robustly restores B lymphopoiesis and T lymphopoiesis, increases naive lymphocyte production, and partially normalizes immune composition.

Notably, these benefits are observed even within aged bone marrow environments, indicating that youthful HSCs retain substantial intrinsic lymphoid competence despite exposure to age-altered niches. These findings, therefore, position HSC replacement not as a universal rejuvenation strategy, but as a targeted means to bypass key intrinsic or niche-associated bottlenecks imposed by aging.

Taken together, replacement-based approaches reinforce a broader conceptual shift toward aligning therapeutic strategy with the primary source of lymphoid failure, whether HSC intrinsic dysfunction, inflammatory suppression, or system-level niche collapse. In this framework, combining lineage-biasing interventions with selective, nongenotoxic replacement may restore clinically meaningful lymphopoiesis without requiring full reversal of hematopoietic aging (Fig. 2).

## CONCLUSION

Single-cell atlases spanning the human lifespan reveal progressive, age-specific remodeling of CD34^+^ HSPC states, characterized by contraction of lymphoid-associated programs and expansion of myeloid-associated and stress-associated populations [37]. In parallel, clonal epigenetic lineage-tracing approaches demonstrate that human hematopoiesis becomes increasingly oligoclonal with age, driven by the dominance of functionally biased clones rather than exhaustion of the HSC pool [38^▪▪^,^39^]. Together, these observations indicate that key organizing principles identified in experimental systems, including cell-state heterogeneity, clonal dominance, and sensitivity to inflammatory and niche-derived cues, are conserved in human hematopoietic aging.

From a translational perspective, these insights argue against uniform rejuvenation strategies and instead favor explicit identification of the dominant bottleneck constraining lymphoid output in each biological or clinical context. In some settings, chronic inflammatory niche signaling may represent the primary suppressive force, favoring interventions that target cytokine pathways or niche-derived ligands (Fig. 2). In others, altered HSPC composition may predominate, motivating strategies that selectively correct dysfunctional states or stabilize lymphoid-competent subsets (Fig. 2). Where system-level constraints prevail, targeted replacement approaches may be required to bypass aged or damaged hematopoietic environments.

Accordingly, the next phase of the field is likely to be defined less by the search for a single rejuvenating intervention than by context-aware deployment of complementary strategies matched to the specific biological barrier limiting lymphopoiesis. Anchoring mechanistic insight to human-relevant measurements and outcomes provides a realistic and tractable path toward restoring immune competence without necessitating complete reversal of hematopoietic aging.

## Acknowledgements

*ChatGPT (OpenAI) was used to aid language editing and stylistic editing. All scientific content, interpretations, and conclusions are the responsibility of the authors.*

### Financial support and sponsorship

*This work was supported by grants to D.B. from the Swedish Childhood Fund (PR2025-0096), the Swedish Cancer Society (243386 Pj), and the Swedish Research Council (2022-00932). A.K.-C. is supported by an EMBO Postdoctoral Fellowship (ALTF 1178-2024).*

### Conflicts of interest

*There are no conflicts of interest.*

## REFERENCES AND RECOMMENDED READING

Papers of particular interest, published within the annual period of review, have been highlighted as:

▪ of special interest
▪▪ of outstanding interest
